# Effects of types and amounts of stabilizers on physical and sensory characteristics of cloudy ready-to-drink mulberry fruit juice

**DOI:** 10.1002/fsn3.206

**Published:** 2015-02-26

**Authors:** Suthida Akkarachaneeyakorn, Sirikhwan Tinrat

**Affiliations:** 1 Department of Agro-Industrial, Food, and Environmental Technology, Faculty of Applied Science King Mongkut's University of Technology North Bangkok1518 Pracharat I Road, Wongsawang, Bangsue, Bangkok, 10800, Thailand; 2 Department of Biotechnology, Faculty of Applied Science, King Mongkut's University of Technology North Bangkok1518 Pracharat I Road, Wongsawang, Bangsue, Bangkok, 10800, Thailand

**Keywords:** Anthocyanin, cloudy mulberry fruit juice, CMC, stabilizer, xanthan gum

## Abstract

In this study, the pH of mulberry juice was optimized for high anthocyanin content and an attractive red color. Mulberry juice pH values of 2.5, 4.0, 6.0, and 8.0 were evaluated. A pH of 2.5 gave an anthocyanin content of 541.39 ± 106.43 mg of cyanidin-3-glucoside per liter, and the a* value was 14 ± 1.00. The effects of stabilizers (CMC and xanthan gum) on the physical characteristics of cloudy ready-to-drink mulberry fruit juice (via the addition of mulberry fruit pulp at a mass fraction of 5%) during storage (4°C for 1 week) were also determined using different mass fractions of the stabilizers (0.1%, 0.3%, and 0.5%). Increasing the stabilizer mass fraction increased the viscosity, turbidity, stability of turbidity, and h* value. Using xanthan gum as the stabilizer produced better results for these parameters than CMC. The type of stabilizer and its mass fraction had no effect on most sensory characteristics, including appearance, color, taste, texture, and overall acceptability (*P* ≥ 0.05), but did affect the odor (*P* ≥ 0.05). Xanthan gum stabilizer gave the juice a better odor than CMC. Cloudy mulberry juice containing 0.5% xanthan gum as the stabilizer had the highest acceptance rate among panelists (average acceptance was 6.90 ± 1.37 points) and produced no precipitate during storage.

## Introduction

Mulberries (genus *Morus*) are grown in the north and northeast of Thailand with approximately 979 Rai (equivalent to 157 ha) (DharmIT 2013). Mulberry fruit has levels of sweetness and sourness that are similar to grapefruit (Agriculture Research Development Agency Thailand 2014). Ripe mulberry fruit is dark red to dark purple, and this coloring arises from the presence of anthocyanins. Anthocyanins are antioxidants and antimicrobial substances. As antioxidants, anthocyanins can function as hydrogen donors to free radicals and capture metallic ions to prevent oxidation reactions (Kong et al. 2003). Therefore, these pigments can reduce the risk of various chronic diseases, such as cancer, diabetes, and coronary thrombosis (Lazze et al. 2004). Mulberry fruit also contains phenol compounds, which are also antioxidants and can prevent inflammation and aneurysms, and hinder the growth of bacteria and viruses (Duthie et al. 2000). Studies have shown that mulberry fruit contains quercetin, which is a flavonoid with antioxidant activity. Quercetin reduces the risk of heart disease and high blood pressure and prevents blood clots (Manach et al. 2005).

Xanthan gum is often used in drinks made with citrus and in fruit-flavored drinks to create a satisfactory texture and as a stabilizer for odor and flavor. The mass fraction of xanthan gum used in these drinks is in the range of 0.001–0.5%. Because it dissolves quickly and completely at low pH, xanthan gum helps with the suspension of insoluble components. It can be mixed with other components in food, including alcohol. Adding a higher mass fraction of xanthan gum (0.025–0.17%) helps to create texture in fruit-flavored drinks (Zecher and Van Coillie 1992). In orange juice, a combination of xanthan gum at 0.02–0.06% and carboxymethyl cellulose (CMC) at 0.02–0.14% is added to aid in the suspension of orange pulp, with CMC stabilizing protein in the orange pulp (Pettitt 1982).

Some fruit juice products with pulp may precipitate after long storage periods, and it is difficult to maintain a homogenous suspension of the fruit pulp. To avoid separation, CMC or mixed hydrocolloids are added to maintain turbidity and suspension. The amount of CMC required for good stability depends on the soluble solid content of the drink and the level of dilution before consumption. Products containing large amounts of soluble solids are viscous and require only a small amount of CMC. By contrast, large amounts of CMC can be used to create texture in products containing few soluble solids. As well as stabilizing fruit pulp, CMC reduces or prevents the formation of oil rings around the neck of the bottle. CMC is added to the product after any preservatives, colors, or flavors. Then, citric acid or other acids can be added to adjust the pH. The mass fraction of CMC that is typically used (0.1–0.4%) provides a moderate to high viscosity. In some cases, CMC is used together with other types of gum (Zecher and Van Coillie 1992).

For fruit juice products that contain a large amount of pulp, it is difficult to stabilize the pulp suspension for long periods. However, the addition of a minimal amount of gum (Anonymous 1981), or an appropriate mixture of natural gums (Ticaloid 550), can yield products with low viscosity and good taste. Additionally, these gums increase the stability of the turbidity of fruit juice stored in bottles. Padival et al. (1980) found that heating fruit juice to inhibit the activity of pectin esterase does not increase the stability of the turbidity in bottled fruit juices with soluble solid contents between 40% and 60%, such as squash and crush. The addition of gum to juices can help resolve these problems.

Mulberry fruit pulp is a source of anthocyanins and other antioxidants. In addition, it provides healthy fiber in mulberry juice. The addition of mulberry fruit pulp to juice reduces waste and increases the product yield of the fruit. To date, there have been no studies on the production of ready-to-drink mulberry juice. Preliminary experiments indicate that the addition of mulberry fruit pulp at a mass fraction of 5% is acceptable to consumers.

This study aims to determine the effect of pH on mulberry juice color and anthocyanin content and the effect of the stabilizer type and amount on the stability of mulberry juice during storage. The effect of stabilizers on sensory characteristics was evaluated for cloudy ready-to-drink mulberry juice stored in glass bottles, which had been pasteurized and stored at 4°C. Two stabilizers, CMC and xanthan gum, were evaluated.

## Materials and Methods

Ripe mulberry fruit (dark purple) from a Chiang Mai breed of mulberry was purchased in Nakorn Chaisri, Nakornpathom Province, Thailand. This fruit had been frozen at −22°C after harvest and was stored at this temperature until analysis. Sodium CMC (BEV 350) and xanthan gum (F80) were provided by Maxway Co., Ltd., Bangkok Thailand. Granulated cane sugar and citric acid were also used in the experiments.

### Properties of the mulberry fruit and pure mulberry juice

The average length, diameter, and weight of the fruit were determined using 30 ripe mulberries.

Pure mulberry fruit juice was prepared by thawing ripe mulberries at room temperature (25°C) and then passing them through a juice extractor three times. The mulberry juice was filtered through cheesecloth on a sieve (100 mesh) and centrifuged at 14000 g for 20 min at 4°C to obtain the juice as the supernatant. The yield, amount of total soluble solids, and the pH of the juice were determined. The amount of anthocyanin (total anthocyanins) was determined using the pH differential method (Giusti and Wrolstad 2005). The results are expressed in milligrams of cyanidin-3-glucoside per liter (mg cyanidin-3-glucoside per L). The total amount of acids (% acidity) in the form of citric acid was determined by titration using a 0.1 mol/L sodium hydroxide solution (AOAC 2012). The color of the juice was recorded as L*, a*, and b* using a Hunter Lab Colorimeter (Color Quest 45/0 Reston, Virginia).

### The effect of pH on color stability and anthocyanin content in pure mulberry juice

The effect of the pH on the color stability and the amount of anthocyanins in pure mulberry juice were studied to find the pH that gave the highest anthocyanin content with an attractive red color.

The pH was adjusted to 2.5, 4.0, 6.0, and 8.0 using a citrate-phosphate buffer solution. Then, the juice was heated to 70°C in a water bath for 5 min. After cooling, the amount of anthocyanin (total anthocyanin) was determined (Giusti and Wrolstad 2005). The color of the cooled juice was described using the L*, a*, b*, and h* value using a Hunter Lab Colorimeter (Color Quest 45/0).

### The effect of stabilizer type and amount on mulberry juice during storage

Fresh mulberry juice was prepared as described in Properties of the mulberry fruit and pure mulberry juice, and the pH was adjusted to the optimum value (The effect of pH on color stability and anthocyanin content in pure mulberry juice) using citric acid. Sugar syrup (glucose) was added until the juice had a total amount of soluble solids of 22°Brix. The mulberry pulp obtained from centrifugation during the juicing process was added to the juice at a mass fraction of 5%. Then, CMC (0.1%, 0.3%, or 0.5%) or xanthan gum (0.1%, 0.3%, or 0.5%) was added as the stabilizer. The mixture (total volume 150 mL) was blended using a magnetic stirrer and placed in a 200-mL glass bottle that had been sterilized with hot water. Once filled, each bottle was heated to 70°C in a temperature-controlled basin for 5 min, cooled, and then stored at 4°C for 1 week. On days 0, 3, and 7, the viscosity was evaluated using a Brookfield viscometer (RVDV-I+, Scientific Promotion Co., Ltd. Stoughton, Massachusetts), and every day the turbidity was determined using a turbidity meter (2100P, HACH). Every day, the appearance of the juice was recorded, and the stability of the turbidity (Padival et al. 1980) was determined by measuring the ratio of the height of fruit pulp (*H*_sediment_) to the height of juice (*H*_total_).

### Sensory evaluation

Consumer acceptability was studied with 20 untrained panelists ranging from 18 to 24 years of age. They were recruited from Faculty of Applied Science, KMUTNB, Bangkok, Thailand. Prior to the sensory evaluation, cloudy mulberry juice samples were refrigerated and served in clear glasses (40 mL glass) topped with transparent, vented lids at 4°C along with nonsalted crackers and distilled water. The evaluation was carried out for mulberry juice with flesh (control) and two samples of mulberry juice: formula 1 was cloudy mulberry juice containing 0.5% CMC as a stabilizer and formula 2 contained 0.5% xanthan gum. The samples were three-digit coded and randomly offered to the panelists. Acceptance testing was used to determine how much each sample was liked based on a 9-point hedonic scale for a set of attributes: appearance, color, odor, taste, texture, and overall acceptability where 9 = like extremely and 1 = dislike extremely.

### Statistical analysis

The evaluation of the effect of pH was carried out using a complete randomized design. All analytical measurements were carried out in triplicate. Data were analyzed using SPSS 20 (SPSS Inc., Armonk, New York). The differences among the pH values were compared using the average values and the least significant difference test at a confidence level of 95%.

The effect of the stabilizer was investigated using a factorial experimental design. All analytical measurements were carried out in triplicate. The data were analyzed using SPSS 20 (SPSS Inc.). The differences among the stabilizers were compared using the average values with Duncan's new multiple range test at a confidence level of 95%.

The sensory evaluation was carried out using a complete randomized design with triplicates. Analysis of variance was conducted on the sample means for appearance, color, odor, taste, texture, and overall acceptability using SPSS 20 (SPSS Inc.). Statistically significant attributes were further analyzed to see where mean differences existed using Least Significant Difference (LSD) at a confidence level of 95%.

## Results and Discussion

### Properties of mulberry fruit and pure mulberry juice

The average length, diameter, and weight of the ripe mulberries used in this study were 18.94 ± 1.72 mm, 9.92 ± 0.66 mm, and 1.39 ± 0.18 g, respectively.

The pure mulberry juice produced from the ripe fruit had 16.00 ± 0.00° Brix of soluble solids. This is similar to the values for Turkish mulberries found by Ercisli and Orhan (2007) (15.90–20.40° Brix). The pH of the juice was 4.12 ± 0.00. The yield of juice from the mulberries was 4.24 ± 1.40%. This yield is low because mulberry fruit is small with low water content. Moreover, the process of juice production has several steps that results in the loss of product. The total initial content of anthocyanin in the mulberry juice was 557.03 ± 0.54 mg of cyanidin-3-glucoside per L. The anthocyanin content in mulberries changes as the fruit ripens, and the content correlates to the color of the fruit. When the mulberry fruit is young, it is green, and has a total anthocyanin content of 2.6–6.8 mg of cyanidin-3-glucoside per L (Hunjaroen and Tongchitpakdee 2010). As the fruit ripens, it turns pink, red, and then dark purple, with the development of a red color associated with the accumulation of anthocyanin and the degeneration of chlorophyll (Mozetic et al. 2004). The total acid content of the juice was 0.68 ± 0.00 g of citric acid/100 mL. For the color coordinates, L* was 1.97 ± 0.59, a* was 0.40 ± 0.03, and b* was −0.57 ± 0.18. The mulberry juice appeared crimson red.

### Effect of pH on color stability and the anthocyanin content in pure mulberry juice

From Table1, when the pH of the juice was increased, the anthocyanin content decreased. Consequently, the anthocyanin contents at pH 2.5 and 4.0 were significantly higher than at pH 6.0 or 8.0 (*P *< 0.05). There was not a significant difference between the anthocyanin contents at pH 2.5 (541.39 ± 106.43 mg of cyanidin-3-glucoside per L) and 4.0 (434.15 ± 88.08 mg of cyanidin-3-glucoside per L) (*P *< 0.05). Similarly, there was not a significant difference between the anthocyanin contents at pH 6.0 (1.76 ± 0.74 mg of cyanidin-3-glucoside per L) and pH 8.0 (59.82 ± 25.79 mg of cyanidin-3-glucoside per L) (*P* ≥ 0.05). These results show that more acidic mulberry juice (lower pH) will have a higher anthocyanin content than less acidic mulberry juice (higher pH). These differences likely arise because structurally anthocyanin is more stable under acidic conditions than neutral or alkaline conditions (Markakis 1982; Bae and Suh 2007). In the present study, the anthocyanin in the mulberry juice at pH 2.5 was the most stable and took the longest time to degrade during heating. These results agree with those of Kirca et al. (2007), who found that the degradation of anthocyanin from black carrots increased as the pH increased. At pH values <2, anthocyanin is in the form of flavylium cations (red), which are stable. When the pH increases, the flavylium cations become unstable and transform to colorless pseudobases at pH 4–5, quinoidal bases (blue) at pH 6–7, and chalcone (light yellow–colorless) at pH values >7 (Markakis 1982; Stintzing and Carle 2004; Kulling 2007).

**Table 1:** Effect of pH on the anthocyanin content in pure mulberry juice

pH values	Monomeric anthocyanin (mg of cyanidin-3-glucoside/L)	Color values, h^*^ (degree)
2.5	541.39 ± 106.43^a^	14.31 ± 1.28^bc^
4.0	434.15 ± 88.08^a^	9.10 ± 2.27^c^
6.0	1.76 ± 0.74^b^	25.51 ± 8.23^b^
8.0	59.82 ± 25.79^b^	65.43 ± 2.72^a^

Data are average values ± standard deviation (*n *= 3). Different letters in columns indicate significant differences at the 95% confidence level.

At pH 8.0, the anthocyanin content is less than that at pH 2.5 or 4.0 because most anthocyanins at this pH are present as chalcone, which is unstable (Markakis 1982). Chalcone degrades quickly and reduces the anthocyanin content compared with the other pH values.

Anthocyanin can be destroyed by heat during the manufacturing process and while in storage. Markakis (1982) reported that heating strawberries at 100°C for 1 h degrades the anthocyanin with a half-life of 1 h. Kirca and Cemeroglu (2003) studied the stability of the anthocyanin in blood oranges at 70, 80, and 90°C and found that the stability of anthocyanin and its half-life decreased as the temperature increased. Kirca et al. (2007) studied the stability of anthocyanin obtained from black carrots in a citrate–phosphate buffer solution at pH 2.5–7.0 with heating to 70, 80, and 90°C. They found that the stability of anthocyanin at the same pH decreased as the temperature increased. These studies all indicate that anthocyanin pigments in vegetables and fruits can be easily destroyed during food processing that incorporates high temperatures, sugar concentrations, pH, amino acid, and ascorbic acid concentrations, and for conditions where oxygen is present. All these conditions could accelerate the degradation of anthocyanin (Kirca et al. 2007). For example, the color of strawberry jam changes from red to red-brown when stored at room temperature for 2 years because of phlobaphene formation.

Color can be described using color space coordinates such as h*, which specifies the color location in degrees (Choudhury 2014). In the present study, the maximum value of h* was obtained at pH 8.0, followed by pH 6.0, 2.5, and 4.0. The h* values at pH 2.5, 4.0, and 6.0 were significantly different than that at pH 8.0 (*P *< 0.05). However, the h* values at pH 2.5, 4.0 and 6.0 were not significantly different from each other (*P* ≥ 0.05). The mulberry juice at pH 2.5, 4.0, and 6.0 was red, while that at pH 8.0 (h* = 65.43 ± 2.72°) was yellowish-red. This corresponds to the theory that at pH values <2, anthocyanin is in the form of flavylium cations (red), which are stable. When the pH increases, the flavylium cations become unstable and transform to colorless pseudobases at pH 4–5, quinoidal bases (blue) at pH 6–7, and chalcone (light yellow–colorless) at pH > 7 (Markakis 1982; Stintzing and Carle 2004; Kulling 2007).

### Effect of the stabilizer type and amount on mulberry juice during storage

#### Changes in the viscosity during storage

**Figure 1:**
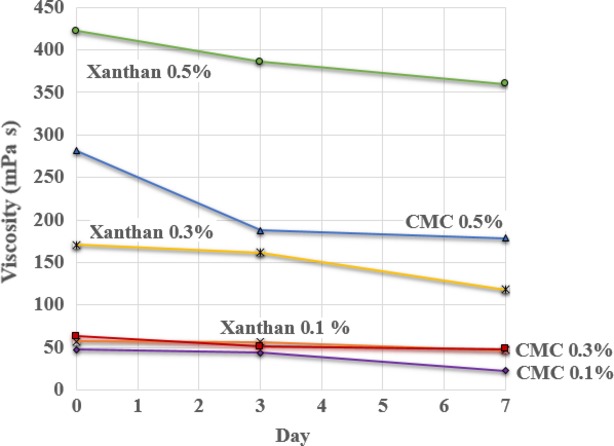
Changes in the viscosity of mulberry juice stabilized with CMC or xanthan gum.

The results for the viscosity of the mulberry juice are shown in Figure1. The viscosity of the mulberry juice was in the range of 22.58–422.11 mPa·s. The storage period, mass fraction of the stabilizer, and type of stabilizer all significantly affected the viscosity (*P *< 0.05). As the mass fraction of the stabilizer increased, the viscosity of the mulberry juice also increased. By contrast, as the storage time increased, the viscosity decreased. These results agree with those of Pangborn et al. (1978), who found that increasing the concentration of gum in drinks caused directly proportional increases in the physical viscosity and its sensory perception. For the mass fraction of stabilizer in the juice, 0.5% CMC gave the highest viscosity (281.33 mPa·s) among the CMC mass fractions tested. Compared with CMC, xanthan gum gave a more viscous product (422.11 mPa·s). Xanthan gum is a branched hydrocolloid with more branches and longer branches than those in the other types of gum, which means it can form many hydrogen bonds and greatly increase the viscosity. With 0.1% and 0.3% CMC, the product had low viscosity, and after leaving to settle for some time, separation of the product occurred. Increasing the mass fraction of CMC in the product can increase the viscosity because of its dependence on the degree of polymerization of CMC. With a high degree of polymerization, the solution viscosity will be high. CMC solutions have similar characteristics to pseudoplastic fluids. By comparison, CMC with a low degree of polymerization will yield solutions with low viscosity that are less like pseudoplastic fluids. If the mass fraction of CMC in the solution is high, the solution viscosity will increase because of the basic properties of hydrocolloids and gums on dissolution and dispersion in water. In addition, because gums are large charged molecules with bulky configurations, they can form hydrogen bonds and reduce the movement of water and flow of a liquid (Szczesniak 1986).

In cloudy mulberry juice products, the addition of 0.5% xanthan gum as a stabilizer will result in a product with high viscosity. By contrast, the addition of 0.1% CMC will result in a product with low viscosity. This means that mulberry juice with 0.1% CMC as the stabilizer will be less stable than that with 0.5% xanthan gum as the stabilizer. This corresponds to the theory of Tan (1990), who found that increasing viscosity corresponds to an increase in the stability of turbidity.

#### Changes in the turbidity during storage

**Figure 2:**
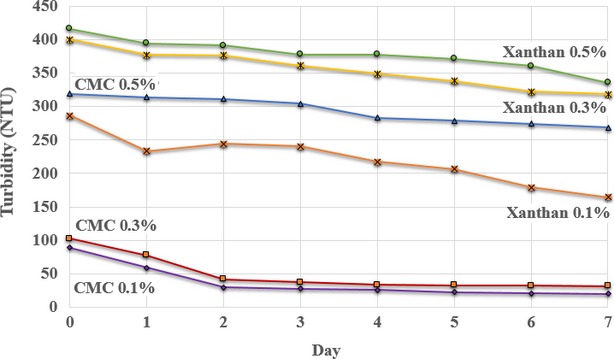
Changes in the turbidity of mulberry juice during storage.

The turbidity of the mulberry juice was studied during storage at 4°C for 1 week (Fig.2 and Table2).

**Table 2:** Stability of the turbidity in cloudy mulberry juice stored at 4°C for 1 week

Type of stabilizer	Mass fraction of stabilizer (%)	Physical stability of turbidity (%)							
		Day 0	Day 1	Day 2	Day 3	Day 4	Day 5	Day 6	Day 7
CMC	0.1	–	–	48	48	48	48	48	48
	0.3	–	–	28	28	28	29	29	32
	0.5	–	–	–	–	–	–	–	–
Xanthan gum	0.1	–	–	–	–	–	–	–	–
	0.3	–	–	–	–	–	–	–	–
	0.5	–	–	–	–	–	–	–	–

–, means no separation of layers. Physical Stability of Turbidity = *H*_sediment_/*H*_total_^*^100%.

The storage period, mass fraction of the stabilizer, and type of stabilizer all significantly (*P *< 0.05) affected the turbidity. In the stabilized juice, as the storage time increased, the stability of the fruit pulp suspension decreased and the turbidity decreased (Fig.2). Observation of precipitate formation and measurement of the height of the precipitate was used to calculate the stability of turbidity as a percentage. The stability of turbidity tended to increase as the storage time lengthened, which suggests that the products separate more over time. Products that have separated have lower turbidity because they are more transparent to light than products with less separation. Therefore, the turbidity decreased as separation of the product increased.

Increasing the mass fraction of the stabilizer affected the turbidity, with higher mass fractions of stabilizer improving stability. Juice stabilized with 0.5% xanthan gum had a high turbidity (416.33 ± 10.02 NTU), which suggests good stability (Fig.2).

When considering the relationship between viscosity and turbidity, products with higher viscosity also had higher turbidity. This suggests that more viscous juices have more stable turbidity than less viscous juices. For both stabilizers, while the addition of more stabilizer increased the stability, it also increased the viscosity. When choosing a stabilizer, the effect it has on the viscosity and the turbidity must be taken into consideration. In particular, the viscosity can affect the acceptance of the product by consumers. While the addition of a stabilizer may improve the stability, if it is highly viscous, it may not be acceptable to consumers.

After storing the mulberry juice that was stabilized with xanthan gum for 1 week, the turbidity was stable with no precipitate (Table2). This result was better than that for the juice with the CMC as the stabilizer, because the solutions stabilized with xanthan gum have higher viscosity than those stabilized with CMC. Although the mass fraction of xanthan gum in the juice was low, xanthan gum is highly stable when heated and has a stable pH. The viscosity of a solution containing xanthan gum will be stable at temperatures in the range of 0–100°C and pH values in the range of 1–13. By comparison, solutions containing CMC solutions will be stable at pH values between 4 and 10, but with the highest viscosity and best stability at pH 7–9. The viscosity of a CMC-stabilized solution will decrease when the pH decreases and the temperature increases. At pH values <3, CMC will be in the form of free acids and will precipitate, while at pH values >10, the solution viscosity will be low. Therefore, in the present study, precipitation will occur in the mulberry juice with CMC as the stabilizer because the pH is <3.

Rothschild and Karsenty (1974) studied the loss of turbidity during storage of pasteurized orange juice and concentrated orange juice. They found that the loss of turbidity during storage resulted from high acidity and/or the activity of any remaining pectin esterase. Both these factors can be resolved by storing the products in cool conditions.

#### Changes in the physical characteristics during storage

When the appearance of the mulberry juice was checked each day for 1 week, a precipitate was observed on the second day of storage for juices with CMC mass fractions of 0.1% and 0.3% (Table2). By contrast, when the CMC mass fraction was increased to 0.5%, no precipitate was observed throughout the 1-week storage. This suggests that the juice containing 0.5% CMC has good stability. With xanthan gum as the stabilizer, no precipitate was observed after storage for 1 week with 0.1%, 0.3%, or 0.5% stabilizer mass fractions. Therefore, xanthan gum provides better stabilization to mulberry juice than CMC, and juice containing xanthan gum could be more acceptable to consumers because of the lack of a precipitate.

### Sensory evaluation

The results of a sensory evaluation using the 9-point hedonic scale are shown in Table3.

**Table 3:** Sensory evaluation results for mulberry juice

Attribute^1^	Cloudy mulberry fruit juice products	
	Formula 1	Formula 2
Appearance^ns^	6.60	6.75
Color^ns^	6.80	7.00
Odor	5.65^b^	6.10^a^
Taste^ns^	6.80	6.55
Texture^ns^	6.10	6.40
Overall acceptability^ns^	7.15	6.90

Different letters in a row indicate the differences are statistically significant at the 95% confidence level, while ^ns^ indicates the difference was not statistically significant at the 95% confidence level. Formula 1 was cloudy mulberry juice containing 0.5% CMC as stabilizer, while formula 2 contained 0.5% xanthan gum.

1 9-Point hedonic scale 1 was the lowest and 9 was the highest score.

The results of sensory testing for appearance, color, taste, texture, and overall acceptability showed no significant difference (*P* ≥ 0.05) between the juices containing xanthan gum and CMC. Therefore, the types of stabilizer did not affect these sensory parameters.

However, for odor, there was a significant difference (*P* ≥ 0.05) between the juices containing xanthan gum and CMC. The tasters tended to prefer the juice containing xanthan gum rather than CMC. Therefore, the type of stabilizer does affect the odor. Xanthan gum is often used as an odor and flavor stabilizer in citrus juices and fruit-flavored drinks because it provides a good texture and odor (Enriquez and Flick 1989).

High-quality mulberry juice should have a homogenous distribution of mulberry fruit pulp. In the present study, a 0.5% mass fraction of CMC or xanthan gum provided the most satisfactory results for pulp distribution.

## Conclusion

The optimum pH for mulberry juice was pH 2.5 because this condition produced the maximum anthocyanin content (541.39 ± 106.43 mg of cyanidin-3-glucoside per L) among the pH values tested. This pH also generated the highest a* value (14.00 ± 1.00), and the resulting product was red, which is an acceptable color to consumers.

Increasing the content of the stabilizer increased both the viscosity and the turbidity of the juice. Xanthan gum provided a better viscosity, turbidity, stability of turbidity, and color (L*, a*, and b* value) in the juice than CMC. In addition, the products containing xanthan gum did not precipitate while stored for 1 week.

The type and amount of stabilizer had no effect on the appearance, color, taste, texture, and overall acceptability of the juice, but it did affect the odor. The xanthan gum imbued the juice with a more acceptable odor than the CMC.

High-quality cloudy mulberry juice should contain a homogenous suspension of mulberry fruit pulp with no precipitate after storage. The xanthan gum stabilizer (mass fraction 0.5%) gave a product that showed no precipitate after storage for 1 week. This product had an average acceptance of 6.90 ± 1.37 points.
